# Non-linear Analysis of Scalp EEG by Using Bispectra: The Effect of the Reference Choice

**DOI:** 10.3389/fnins.2017.00262

**Published:** 2017-05-16

**Authors:** Federico Chella, Antea D'Andrea, Alessio Basti, Vittorio Pizzella, Laura Marzetti

**Affiliations:** ^1^Department of Neuroscience, Imaging and Clinical Sciences, G. d'Annunzio University of Chieti-PescaraChieti, Italy; ^2^Institute for Advanced Biomedical Technologies, G. d'Annunzio University of Chieti-PescaraChieti, Italy

**Keywords:** EEG reference, EEG functional connectivity, non-linear connectivity, bispectral analysis, bicoherence, antisymmetric cross-bispectrum

## Abstract

Bispectral analysis is a signal processing technique that makes it possible to capture the non-linear and non-Gaussian properties of the EEG signals. It has found various applications in EEG research and clinical practice, including the assessment of anesthetic depth, the identification of epileptic seizures, and more recently, the evaluation of non-linear cross-frequency brain functional connectivity. However, the validity and reliability of the indices drawn from bispectral analysis of EEG signals are potentially biased by the use of a non-neutral EEG reference. The present study aims at investigating the effects of the reference choice on the analysis of the non-linear features of EEG signals through bicoherence, as well as on the estimation of cross-frequency EEG connectivity through two different non-linear measures, i.e., the cross-bicoherence and the antisymmetric cross-bicoherence. To this end, four commonly used reference schemes were considered: the vertex electrode (Cz), the digitally linked mastoids, the average reference, and the Reference Electrode Standardization Technique (REST). The reference effects were assessed both in simulations and in a real EEG experiment. The simulations allowed to investigated: (i) the effects of the electrode density on the performance of the above references in the estimation of bispectral measures; and (ii) the effects of the head model accuracy in the performance of the REST. For real data, the EEG signals recorded from 10 subjects during eyes open resting state were examined, and the distortions induced by the reference choice in the patterns of alpha-beta bicoherence, cross-bicoherence, and antisymmetric cross-bicoherence were assessed. The results showed significant differences in the findings depending on the chosen reference, with the REST providing superior performance than all the other references in approximating the ideal neutral reference. In conclusion, this study highlights the importance of considering the effects of the reference choice in the interpretation and comparison of the results of bispectral analysis of scalp EEG.

## 1. Introduction

How synchronization affects communication between groups of neurons represents one of the central issues of neuroscience. Several studies have been conducted to investigate neuronal functional communication, postulating a model of the human brain as a complex integrated system (Fries, 2005, 2015; Friston, 2011). It is now clear that, among the different neuroimaging techniques, electroencephalography (EEG) can be considered as an excellent tool for the study of neuronal interactions in both research and clinical practice (Friston and Frith, 1995; Stam et al., 2007; Fogelson et al., 2013; Frantzidis et al., 2014). Indeed, thanks to its high temporal resolution, EEG can provide insights into coupling of cortical oscillations hypothesized as the mechanism that underpins local and long-range neuronal communication (Tallon-Baudry et al., 1996; Womelsdorf and Fries, 2006; Fries, 2015).

In this framework, local and long-range synchronization can occur either in selected frequency bands (e.g., Palva and Palva, 2007; Hipp et al., 2012; Engel et al., 2013; Marzetti et al., 2013) or in a more sophisticated fashion which involves the interaction between different frequencies, i.e., non-linear synchronization. The latter possibly serves as a carrier mechanism for the integration of spectrally distributed processing (Varela et al., 2001; Palva et al., 2005; Jensen and Colgin, 2007; Canolty and Knight, 2010), providing a plausible physiological mechanism for linking activity at different temporal rates. Bispectral analysis has proven to be an effective tool to assess non-linear synchronization in human EEG (Dumermuth et al., 1971; Sigl and Chamoun, 1994; Darvas et al., 2009a,b; Chella et al., 2014, 2016b; Özkurt, 2016). Notably, bispectral measures such as bicoherence (Dumermuth et al., 1971; Sigl and Chamoun, 1994) were successfully used to detect non-linear long-range coupling from scalp EEG data in healthy subjects (ShilS et al., 1996; Schack et al., 2002; Isler et al., 2008; Chella et al., 2014). Moreover, the information from bispectral analysis of the EEG signals is highly used in clinical applications, such as in the determination of consciousness states and anesthetic depth levels (Freye and Levy, 2005; Pritchett et al., 2010), or in the identification and prediction of epileptic seizures (Bullock et al., 1997; Mormann et al., 2005; Chua et al., 2009). Indeed, changes in scalp EEG bicoherence have been shown to index the effects of the different drugs used to induce clinical anesthesia (Pritchett et al., 2010), as well as to indicate anesthesia vs. conscious states (Pritchett et al., 2010; Hayashi et al., 2014). Moreover, in epileptology the clinical markers of local hypersynchronous activity of the neuronal pools can be identified through information on higher order spectra extracted from EEG data (Chua et al., 2009).

Of note, all the above studies directly rely on bispectral indices derived from channel level EEG data. Nevertheless, since EEG measures only electric potential differences, it is implicitly assumed that signals at a given EEG electrode are referred to a neutral reference. Previous works have investigated different options in the attempt to find a neutral reference location. Several referencing schemes have been suggested like the vertex (Lehmann et al., 1998; Hesse et al., 2004), unimastoid (Başar et al., 1998; Thatcher et al., 2001), linked mastoids (Gevins and Smith, 2000; Croft et al., 2002), or nose (Andrew and Pfurtscheller, 1996; Essl and Rappelsberger, 1998), but no true neutral location has been found (Nunez and Srinivasan, 2006). Moreover, the average reference (Offner, 1950; Nunez et al., 2001) and the Reference Electrode Standardization Technique (REST) (Yao, 2001) have been shown to be valid solutions. Despite the proven advantages of the latter strategies, these are not completely free from biases (Desmedt et al., 1990; Dien, 1998; Zhai and Yao, 2004). Thus, it has to be kept in mind that a non-neutral reference affects the spatial and temporal features of EEG recordings, leading to possible distortions in the results. Recent works, through simulated and real data, provided a quantitative overview of the perturbation generated by the reference choice on the estimation of EEG voltage waveforms or scalp distributions (Joyce and Rossion, 2005; Yao et al., 2007; Tian and Yao, 2013; Liu et al., 2015), spectral power (Yao et al., 2005), correlation and coherence (Andrew and Pfurtscheller, 1996; Essl and Rappelsberger, 1998; Rummel et al., 2007; Müller et al., 2014), and linear functional connectivity (Guevara et al., 2005; Marzetti et al., 2007; Qin et al., 2010; Chella et al., 2016a).

To date, despite the wide use of bispectral analysis in EEG as above documented, no quantification of the effects of the use of different referencing schemes on these indices has been provided. The aim of this paper is to provide such quantification through simulated and real data, for local synchrony assessment through bicoherence as well as for long range synchrony characterization through two different non-linear metrics: (i) cross-bicoherence, (ii) antisymmetric cross-bicoherence. To this end, the vertex electrode (Cz), the digitally linked mastoids, the average reference, and the REST transformation were considered and different electrode densities were taken into account. In addition, the effects of the accuracy in the head model used to build the REST transformation have been assessed.

## 2. Materials and methods

### 2.1. Theoretical background

#### 2.1.1. Bispectral analysis

This subsection recalls the basic principles and properties of bispectral analysis of EEG signals used in this study. Let, *v*_*i*_ be the time series of the signal recorded by the *i*th EEG channel. The auto-bispectrum of *v*_*i*_ can be estimated as (Nikias and Petropulu, 1993):

(1) $$\begin{array}{r} B_{i}\left(f_{1},f_{2}\right)=\langle {\hat{\upsilon }}_{i}\left(f_{1}\right){\hat{\upsilon }}_{i}\left(f_{2}\right){\hat{\upsilon }}_{i}^{*}\left(f_{1}+f_{2}\right)\rangle \end{array}$$

where ${\hat{\upsilon }}_{i}\left(f_{1}\right)$, ${\hat{\upsilon }}_{i}\left(f_{2}\right)$, and ${\hat{\upsilon }}_{i}\left(f_{1}+f_{2}\right)$ are the Fourier coefficients of the signal components at frequencies *f*_1_, *f*_2_, and *f*_1_ + *f*_2_, and the symbols ^*^ and 〈 · 〉 denote the complex conjugation and the expectation value, respectively. In practice, the expectation value is replaced by the average over a sufficiently large number of signal realizations, or data segments. The auto-bispectrum of a signal is a measure of the non-linear cross-frequency coupling between signal components at three different frequencies, i.e., *f*_1_, *f*_2_, and *f*_3_ = *f*_1_ + *f*_2_. In particular, the third frequency is set to the sum of the other two because all the other choices lead to vanishing bispectra for stationary processes, or also for non-stationary processes if the experimental design is not appropriate (Chella et al., 2016b). The non-linear coupling essentially means the synchronization of the phases of the above frequency components, i.e., φ_*i*_(*f*_1_), φ_*i*_(*f*_2_), and φ_*i*_(*f*_1_ + *f*_2_), in such a way that the generalized phase difference Δφ_*i*_ = φ_*i*_(*f*_1_) + φ_*i*_(*f*_2_)−φ_*i*_(*f*_1_ + *f*_2_) stays close to a constant value. This kind of interaction is usually termed quadratic phase coupling (Kim and Powers, 1978; Nikias and Petropulu, 1993).

The auto-bicoherence, simply referred to as bicoherence in this study, is the normalized version of the auto-bispectrum in Equation (1), i.e.,

(2) $$\begin{array}{r} b_{i}\left(f_{1},f_{2}\right)=\frac{B_{i}\left(f_{1},f_{2}\right)}{N_{i}\left(f_{1},f_{2}\right)} \end{array}$$

with $N_{i}\left(f_{1},f_{2}\right)$ being a normalization factor. There are a number of expressions for bicoherence (Brillinger, 1965; Kim and Powers, 1979; Hinich and Wolinsky, 2005; Helbig et al., 2006), which essentially differ only by the normalization factor used. In the present study, the normalization factor suggested by Shahbazi et al. (2014) is used, i.e.,

(3) $$\begin{array}{r} N_{i}\left(f_{1},f_{2}\right)=Q_{i}\left(f_{1}\right)Q_{i}\left(f_{2}\right)Q_{i}\left(f_{1}+f_{2}\right) \end{array}$$

with

(4) $$\begin{array}{r} Q_{i}\left(f\right)={\left(\frac{1}{L}\underset{l}{\sum }|{\hat{\upsilon }}_{i}\left(f,l\right){|}^{3}\right)}^{1/3} \end{array}$$

being [apart a multiplicative factor (1/*L*)^1/3^] the three-norm of a *L*-length vector ${\hat{\upsilon }}_{i}\left(f,l\right)$, whose elements are the Fourier coefficients of the signal in channel *i* at frequency *f* estimated from the segment *l*. Of note, this normalization factor guarantees that the magnitude of the bicoherence is bounded between 0 (i.e., no interaction) and 1 (i.e., maximum interaction).

Following the definition of bicoherence, the cross-bicoherence is used to determine the non-linear phase synchronization between the frequency components of signals measured at three different channels, i.e., *v*_*i*_, *v*_*j*_, and *v*_*k*_, and it reads:

(5) $$\begin{array}{r} cb_{ijk}\left(f_{1},f_{2}\right)=\frac{B_{ijk}\left(f_{1},f_{2}\right)}{N_{ijk}\left(f_{1},f_{2}\right)}=\frac{\langle {\hat{\upsilon }}_{i}\left(f_{1}\right){\hat{\upsilon }}_{j}\left(f_{2}\right){\hat{\upsilon }}_{k}^{*}\left(f_{1}+f_{2}\right)\rangle }{Q_{i}\left(f_{1}\right)Q_{j}\left(f_{2}\right)Q_{k}\left(f_{1}+f_{2}\right)}. \end{array}$$

For this reason, the cross-bicoherence is used as a measure of non-linear functional relationships between EEG channels, i.e., a measure of EEG non-linear functional connectivity (ShilS et al., 1996; Schack et al., 2002; Isler et al., 2008).

The estimation of functional connectivity from EEG signals has to face the problem of the artifacts due to volume conduction (Nunez et al., 1997; Nolte et al., 2004; Srinivasan et al., 2007). These are essentially due to the widespread representation of brain source activity over the scalp and are especially relevant for nearby channels (Winter et al., 2007; Schoffelen and Gross, 2009). For instance, two EEG channels can record, with some weights, from the same neural population, opening the possibility for spurious interactions between channels even in the absence of actual brain interactions. Almost all the measures of linear and non-linear connectivity, including the cross-bispectra and the cross-bicoherence, are sensitive to these artifacts (Schoffelen and Gross, 2009). In order to address this problem in relation to bispectral analysis of EEG signals, in Chella et al. (2016b) it has been suggested to use the antisymmetric cross-bicoherence, i.e.,

(6) $$\begin{array}{r} acb_{ijk}\left(f_{1},f_{2}\right)=\frac{B_{ijk}\left(f_{1},f_{2}\right)-B_{kji}\left(f_{1},f_{2}\right)}{N_{ijk}\left(f_{1},f_{2}\right)+N_{kji}\left(f_{1},f_{2}\right)} \end{array}$$

namely the normalized difference between two cross-bispectra where two of the channel indices have been switched. Indeed, this quantity cannot be generated by a superposition of independent sources and, thus, necessarily reflects genuine brain interactions as opposite to the artifacts due to volume conduction (Chella et al., 2014).

Finally, it can be noted that the bicoherence, the cross-bicoherence, and the antisymmetric cross-bicoherence are complex-valued quantities. In this paper, however, in order to be interpreted as indices of non-linear properties and functional relationships of the EEG signals, these quantities will be considered in magnitude.

#### 2.1.2. EEG reference schemes

In this subsection, notations and formulas for the re-referencing transformations used in this paper are introduced. Let *V*_*m*_ be a *N* × *M* matrix, with *N* being the number of channels and *M* being the number of time samples, containing the EEG recordings measured by using a given reference scheme. The re-referencing to a different EEG reference scheme, here generically labeled as X, can be performed by using the following transformation:

(7) $$\begin{array}{r} V_{\text{X}}=V_{m}-V_{{\text{ref}}_{\text{X}}}=T_{\text{X}}V_{m} \end{array}$$

where *V*_X_ is the matrix containing the re-referenced EEG recordings, *V*_ref_X__ is the matrix containing *N* copies of the reference signal, and *T*_X_ is a *N* × *N* transformation matrix.

Along this line, the reference to the physical electrode Cz is obtained by subtracting from each channel and for each time sample the potential measured at Cz. The corresponding transformation matrix is:

(8) $$\begin{array}{r} T_{\text{Cz}}=𝕀-R_{\text{Cz}} \end{array}$$

with 𝕀 being the *N* × *N* identity matrix, and *R*_Cz_ being a *N* × *N* matrix with all the elements equal to 0 except for those of the column corresponding to the Cz channel, which are equal to 1.

The reference signal for the digitally linked mastoid (DLM) reference is the average between the signals recorded at the electrodes located over (or in proximity of) the left and right mastoids. Then, the re-referencing transformation can be written as:

(9) $$\begin{array}{r} T_{\text{DLM}}=𝕀-R_{\text{DLM}} \end{array}$$

with *R*_DLM_ having all the elements equal to 0 except for those of the columns corresponding to left and right mastoid channels, which are equal to 0.5.

The average reference (AVE) is performed by subtracting, for each time sample, the average of all the electrodes from each channel. The corresponding transformation matrix is:

(10) $$\begin{array}{r} T_{\text{AVE}}=𝕀-R_{\text{AVE}} \end{array}$$

with *R*_AVE_ having all the elements equal to 1/*N*.

The REST (Yao, 2001) aims at constructing a virtual reference to a point located at infinity. The REST exploits the fact that the EEG potentials measured with any original reference and those referenced to a point at infinity are generated by the same neuronal sources, i.e.,

(11) $$\begin{array}{rcl} V_{m} & = & G_{m}S \end{array}$$

(12) $$\begin{array}{rcl} V_{\text{REST}} & = & G_{\text{REST}}S \end{array}$$

with *S* being the matrix of the source activities, and *G*_*m*_ and *G*_REST_ being the transfer matrices from these sources to EEG sensors, i.e, the lead field matrices. Since the inverse problem solution is not affected by the choice of the EEG reference, at least for noiseless potentials (Pascual-Marqui and Lehamann, 1993; Geselowitz, 1998), an estimate of *S* can be obtained by inverting Equation (11), i.e.,

(13) $$\begin{array}{l} S=G_{m}^{+}V_{m} \end{array}$$

with (·)^+^ denoting the Moore-Penrose generalized inverse. Then, by combining Equations (12, 13), the transformation matrix for REST can be derived as follows:

(14) $$\begin{array}{r} T_{\text{REST}}=G_{\text{REST}}G_{m}^{+} \end{array}$$

A key feature of REST is that, since only the transfer matrices *G*_REST_ and *G*_*m*_ are needed to built the transformation matrix, the actual sources *S* do not need to be found explicitly. Indeed, based on the equivalent source technique (Yao, 1996, 2000, 2003), it is sufficient to assume an equivalent source distribution (ESD) and calculate *G*_REST_ and *G*_*m*_ for this ESD rather than for the actual sources. In this study, the ESD was assumed consisting in a discrete layer of current dipoles forming a closed surface, in analogy with previous studies (Yao, 2001; Yao et al., 2005; Marzetti et al., 2007; Chella et al., 2016a). This also has the advantage that the transformation matrix does not depend on the actual data, thus allowing, for instance, to re-reference different sessions of the same EEG acquisition by using the same transformation matrix. However, the transformation matrix still depends on the accuracy of the EEG forward solution in the calculation of the transfer matrices, which in turn depends on a number of choices including, e.g., the volume conductor model, the EEG forward solver, the EEG electrode density or locations. Some of these aspects will be investigated in this paper.

### 2.2. Simulations

The effects of the reference choice on the estimation of non-linear features of scalp EEG data were first assessed by using simulations. Indeed, differently from real world experiments, in simulations it is possible to measure the potential difference between any point over the scalp and a reference point located infinitely far from the head, where the electric field generated by brain sources vanishes, thus allowing to simulate an “ideal” neutral reference and, thus, unbiased EEG recordings. The simulations performed in this work followed an approach similar to the one used in our previous study (Chella et al., 2016a) to assess the changes induced by the EEG reference in linear connectivity patterns of EEG imaginary coherency (Nolte et al., 2004; Marzetti et al., 2008). In brief, in the present work, the analyses of bicoherence and non-linear connectivity based on either cross-bicoherence or antisymmetric cross-bicoherence were performed on various simulated datasets referenced to a point at infinity, as well as on the re-referenced datasets derived from the former by applying each of the reference schemes presented in Section 2.1.2. The effects of the reference choice were then assessed through the comparison between the results obtained prior and after re-referencing, considering as gold standard the results for the datasets referenced to a point at infinity.

#### 2.2.1. Generation of simulated EEG data

Ten realistic head models, with different head shapes to account for inter-subject anatomical variability, were built based on the segmentation of high resolution whole-head anatomical magnetic resonance images (MRIs) acquired from the 10 subjects participating to the real data experiment described in this paper (see Section 2.3.1). The MRI segmentation was performed by using the Curry 6.0 software package (Neuroscan Compumedics USA, Charlotte, NC, USA), and resulted in the generation of triangulated meshes for the boundaries between gray matter and CSF (cortex), CSF and skull (inner skull), skull and skin (outer skull), and for the outer surface of the head (skin). For each head model, a three-shell volume conductor model, i.e., including the brain, the skull and the scalp, was built using the shapes of the inner skull, outer skull, and skin meshes. Conductivities were set equal to 0.33 S/m for the brain and scalp, and 0.0066 S/m for the skull. The source space consisted in a regular grid with 5 mm step inside the volume bounded by the cortical mesh. A 128-channel EEG sensor net was registered to the head models, with the electrodes located at the standard positions of the 10-5 system (Oostenveld and Praamstra, 2001).

Given a set of current dipole sources, 5 min simulated EEG recordings referenced to a point at infinity, sampled at 500 Hz, were generated from source time courses by solving the EEG forward problem. The set of sources included two non-linear coupled sources and four uncorrelated sources of noise, the latter aiming to mimic background brain activity. All of these sources were randomly located and oriented over the source space. The time courses for the two non-linear coupled sources were generated by using a time-delayed interaction model, i.e.,

(15) $$\begin{array}{r} s_{2}\left(t\right)=s_{1}\left(t-\tau \right) \end{array}$$

with *s*_1_ and *s*_2_ being two non-linear sources with quadratic non-linearity, and τ being equal to 10 ms. This model was previously used in Chella et al. (2014, 2016b) for testing the properties of antisymmetric bispectral measures. In the present study, *s*_1_ was generated by summing the time courses of three quadratically phase coupled oscillators centered at 6, 10, and 16 Hz. The former two oscillators were obtained by band-pass filtering two i.i.d. white Gaussian processes around 6 and 10 Hz, respectively. The latter oscillator was generated by a multiplicative interaction (i.e., a time-point by time-point multiplication) between the other two oscillators, followed by filtering around 16 Hz. A Butterworth filter with 1 Hz bandwidth was used for the filtering at the above three frequencies, performing filtering in both the forward and the reverse directions to ensure zero phase distortion. The time courses for the four uncorrelated sources of noise were simulated as broadband white Gaussian processes filtered between 0.5 and 100 Hz.

By using the information about the realistically shaped head model and the EEG electrode locations, the lead field matrix for the simulated sources with the reference to a point at infinity was computed according to Nolte and Dassios (2005). The EEG recordings were then generated by multiplying the time courses of the simulated sources with the lead field matrix. The signal-to-noise ratio (SNR) was set equal to 1, with the SNR being defined as the ratio between the mean variance across channels of the signals generated by the interacting sources and the mean variance of the signals generated by the sources of noise. A low level of uncorrelated white Gaussian noise was also added to sensor signals to mimic instrumental noise. One-hundred different dataset were generated for each of the 10 realistic head models by randomizing source locations and orientations, resulting in a total amount of 1,000 different dataset on which the various reference schemes were tested.

#### 2.2.2. Re-referencing of simulated EEG recordings

From the datasets referenced to a point at infinity, the datasets re-referenced to Cz, DLM, and AVE were obtained by applying the transformations in Equations (8–10). The datasets re-referenced using REST were obtained by applying the transformation in Equation (14) to data previously re-referenced to the physical reference Cz.

To investigate the effectiveness of REST in dependence on the head modeling accuracy, the REST transformation was calculated for three different volume conductor models with increasing complexity levels: (i) a three-concentric-shell *spherical* model (Yao, 2001; Yao et al., 2005; Marzetti et al., 2007; Zappasodi et al., 2014, 2015; Liu et al., 2015; Chella et al., 2016a), whose dimensions were based on the dimensions of a standard head provided by the MNI-152 template (Fonov et al., 2009, 2011); (ii) a three-shell *realistic standard* model (Chella et al., 2014, 2016a) obtained from the segmentation of the MNI-152 template (Fonov et al., 2009, 2011); and (iii) a three-shell *realistic individual* model (Zhai and Yao, 2004; Liu et al., 2015; Chella et al., 2016a) obtained from the segmentation of subject individual MRI. Notably, the latter model was similar but not exactly the same model used for the generation of simulated EEG recordings. Specifically, in order to fulfill independence between the two models, the one used for the REST re-referencing was derived from the one used to generate the EEG datasets after re-sampling of the boundary meshes of head compartments. Tissue conductivities were set to 0.33 S/m for the innermost (brain) and outermost (scalp) compartments, and to 0.0066 S/m for the intermediate compartment (skull). The equivalent source distribution consisted in 4,000 current dipoles uniformly distributed and normally oriented over a closed surface. Specifically, for the spherical model (i.e., case i), the closed surface was formed by a spherical cap closed on the bottom by a transverse plane (Marzetti et al., 2007; Chella et al., 2016a). For the realistic models (i.e., cases ii and iii), the closed surface was constructed by contracting the brain mesh to 95% of its size (Zhai and Yao, 2004). The REST transformations using the spherical, realistic standard and realistic individual models were labeled as REST_sph_, REST_std_, and REST_ind_, respectively.

#### 2.2.3. Bicoherence and cross-bicoherence analysis

The simulated datasets were divided into 1 s non-overlapping segments. Within each segment, data were Hanning windowed, and the Fourier coefficients were evaluated using conventional FFT algorithms. Bicoherence and cross-bicoherence analyses were then restricted to the three frequencies of interest considered for the generation of simulated recordings, i.e., *f*_1_ = 6Hz, *f*_2_ = 10Hz, and *f*_3_ = *f*_1_ + *f*_2_ = 16Hz. The bicoherence *b*_*i*_(*f*_1_, *f*_2_) was estimated for each channel *i* according to Equation (2). Cross-bicoherence *cb*_*ijk*_(*f*_1_, *f*_2_) and antisymmetric cross-bicoherence *acb*_*ijk*_(*f*_1_, *f*_2_) were estimated for each possible triplet of channels denoted by indices *i*, *j* and *k* according to Equations (5, 6), respectively.

#### 2.2.4. Performance measures

To assess the performances of the various reference schemes, the estimates of bicoherence, cross-bicoherence, and antisymmetric cross-bicoherence obtained from the original datasets referenced to a point at infinity were compared to those obtained after re-referencing in terms of relative error (RE) (Yao, 2001; Zhai and Yao, 2004; Marzetti et al., 2007; Liu et al., 2015; Chella et al., 2016a), i.e.,

(16) $$\begin{array}{r} RE_{b}^{\text{X}}=\frac{\sqrt{\underset{i}{\sum }|b_{i}^{\text{X}}-b_{i}^{\text{INF}}{|}^{2}}}{\sqrt{\underset{i}{\sum }|b_{i}^{\text{INF}}{|}^{2}}} \end{array}$$

(17) $$\begin{array}{r} RE_{cb}^{\text{X}}=\frac{\sqrt{\underset{i,j,k}{\sum }|cb_{ijk}^{\text{X}}-cb_{ijk}^{\text{INF}}{|}^{2}}}{\sqrt{\underset{i,j,k}{\sum }|cb_{ijk}^{\text{INF}}{|}^{2}}} \end{array}$$

(18) $$\begin{array}{r} RE_{acb}^{\text{X}}=\frac{\sqrt{\underset{i,j,k}{\sum }|acb_{ijk}^{\text{X}}-acb_{ijk}^{\text{INF}}{|}^{2}}}{\sqrt{\underset{i,j,k}{\sum }|acb_{ijk}^{\text{INF}}{|}^{2}}} \end{array}$$

where the superscript INF denotes the reference to a point at infinity, the superscript X is an alternative among Cz, DLM, AVE, REST_sph_, REST_std_, or REST_ind_, and the subscripts *i*, *j*, and *k* run over 1, …, *N*, with *N* being the number of channels. The contrast between the different EEG reference schemes was performed by looking at the distributions of the RE from all simulation repetitions.

In order to investigate the effects of EEG electrode density, the above analyses were repeated for three subsets of the 128 simulated recordings, corresponding to the following EEG sensor layouts: (i) a 21-channel system, including to the 19 electrodes of the 10–20 International system (Jasper, 1958) with the addition of TP9 and TP10 electrodes; (ii) a 34-channel system, including a selection of the electrodes of the 10–10 system (Chatrian et al., 1985); and (iii) a 74-channel system, i.e., the full 10–10 system (Chatrian et al., 1985).

### 2.3. Real EEG data

A real EEG experiment was carried out to provide an example of the effects of the EEG reference choice in actual experimental situations. In particular, our experiment aimed at evaluating the changes in the patterns of resting state EEG bicoherence and bispectrum-based non-linear connectivity induced by the chosen reference scheme.

#### 2.3.1. Data acquisition and preprocessing

Ten healthy adults subjects (age 20–29 years; gender 2 F, 8 M) were recruited for the experiment. The study was approved by the Ethics Committee for Biomedical Research of the Provinces of Chieti and Pescara and of the G. d'Annunzio University of Chieti-Pescara. All subjects gave written informed consent in accordance with the Declaration of Helsinki. Experiments were performed in a quiet room with soft natural light. Subjects were requested to sit in a comfortable chair, relax and fix a cross in front of them. Measurement consisted of 10 min of continuous eyes-open resting state activity. The EEG signals were recorded using a 128-channel HydroCel GSN net (Electrical Geodesics, Inc., Eugene, OR, USA) referenced to Cz. The electrode impedance was kept below 100 kΩ. Data were sampled at 1 kHz. The locations of EEG electrodes on the scalp and of three anatomical landmarks (nasion, left, and right pre-auricolar points) were measured using a 3D digitizer (Polhemus, Colchester, VT, USA).

For each subject, high resolution whole-head anatomical MRIs were also acquired in order to construct a realistic individual head model for the re-referencing using REST. MRIs were acquired by using a 3 T Philips Achieva scanner (Philips Medical Systems, Best, The Netherlands) via a 3D fast field echo T1-weighted sequence (MP-RAGE; voxel size 1 mm isotropic; repetition time 8.1 ms; echo time 3.7 ms; flip angle 8°; SENSE factor 2). The coregistration of EEG electrode locations with the MRI volume was performed based on the match between anatomical landmark locations identified in the two imaging modalities.

As a preprocessing step, the signals from the electrodes located over the face and neck were excluded from the analysis because highly contaminated by muscular activity. The number of available recording channels was thus reduced to 110. Raw data were band-pass filtered at 0.5–100 Hz and a visual inspection was carried out to remove the segments of signals containing spikes, eye blinks or horizontal movements. An independent component analysis (ICA) was then performed to remove biological and instrumental artifacts. Specifically, ICA was performed by using the FastICA algorithm with deflationary orthogonalization and tanh non-linearity (Hyvärinen and Oja, 2000). The extracted independent components were visually inspected and classified as artifactual components or as components of brain origin on the basis of their topographies, power spectral density and time courses. The independent components classified as artifactual were rejected. Particular attention was paid to the removal of hearth related activity. In order to allow for across-subject averaging or comparison between subjects, a few missing channels (i.e., one channel in 3 out of 10 subjects and two channels in 2 out of 10 subjects), excluded from the set of 110 channels prior to ICA because extremely noisy or damaged, were interpolated from clean signals by using the spherical interpolation method (Perrin et al., 1989) implemented in the FieldTrip software package (Oostenveld et al., 2011).

#### 2.3.2. EEG data re-referencing

The EEG signals were acquired with Cz as a physical reference. The other reference schemes (i.e., DLM, AVE, REST_sph_, REST_std_, or REST_ind_) were applied to preprocessed signals using the transformations in Equations (9, 10, 14). Analogously to the re-referencing of simulated EEG data discussed in Section 2.2.2, the REST transformations were calculated by assuming an equivalent source distribution consisting of 4,000 current dipoles uniformly distributed and normally oriented over a closed surface encompassing the brain volume. Conductivities for the brain, the skull and the scalp were set equal to 0.33, 0.0066, and 0.33 S/m, respectively. Forward solutions based on a spherical, realistic standard or realistic individual three-shell volume conductor model were computed according to Nolte and Dassios (2005).

#### 2.3.3. EEG data analysis

The analysis was first focused on the estimation of EEG bicoherence. Signals were divided into 1 s non-overlapping segments. Within each segment, data were Hanning windowed and Fourier transformed, and the bicoherence *b*_*i*_(*f*_1_, *f*_2_) was estimated for each channel *i* = 1, …, *N* according to Equation (2). The resulting frequency resolution was 1 Hz on both the *f*_1_ and the *f*_2_ axis. This analysis was performed for all the combinations of frequencies (*f*_1_, *f*_2_) up to *f*_1_ + *f*_2_ = 40Hz, but it was then restricted to a single frequency pair corresponding to the individual peak of bicoherence. In particular, a peak of bicoherence was observed at around (*f*_1_, *f*_2_) = (10, 10Hz) in all the subjects (shown in Figure S1 of the Supplementary Material), which reflects a non-linear coupling between EEG signal components in the alpha (*f*_1_ = *f*_2_ = 10Hz) and beta (*f*_3_ = *f*_1_ + *f*_2_ = 20Hz) bands. Scalp distributions of bicoherence at the individual frequency pair and from the different reference schemes were used to assess the effects of the reference choice. Moreover, in order to assess whether the latter affects a global measure of bicoherence, the maximum value of bicoherence over channels was considered for each subject, i.e.,

(19) $$\begin{array}{r} b^{max}=\underset{i\;=\;1,\ldots ,N}{\max}b_{i} \end{array}$$

This index essentially aims at measuring the maximum level of bicoherence regardless of the location over the scalp, and it has been used in some clinical practice, e.g., to investigate the changes related to anesthetic concentration in bicoherence measurements (Hagihira et al., 2002, 2004; Morimoto et al., 2006; Pritchett et al., 2010).

In order to perform connectivity analysis, we chose two seed channels [60 and 85 in the EGI's sensor net, equivalent to P1 and P2 in the 10-10 system (Luu and Ferree, 2005)] overlying the left and right medial-parietal areas, i.e., two regions where the observed bicoherence was rather prominent regardless of the reference scheme used. We then considered the cross-bicoherence and the antisymmetric cross-bicoherence with respect to each of the two seed channels as metrics of interest to evaluate functional connectivity. In particular, the connectivity of a generic channel *i* to the seed channel was assessed by using a bivariate version of Equations (5, 6), i.e., obtained by setting the second channel index equal to the first, as follows:

(20) $$\begin{array}{r} cb_{\text{P}1-i}\left(f_{1},f_{2}\right)≜cb_{\text{P}1\text{ P}1i}\left(f_{1},f_{2}\right) \end{array}$$

(21) $$\begin{array}{r} acb_{\text{P}1-i}\left(f_{1},f_{2}\right)≜acb_{\text{P}1\text{ P}1i}\left(f_{1},f_{2}\right) \end{array}$$

for P1, and similarly for P2.

#### 2.3.4. Group analysis and statistics

Topographical maps of bicoherence, cross-bicoherence, and antisymmetric cross-bicoherence were evaluated for each subject and for each re-referenced dataset separately. Group-level results were obtained by across-subject averaging. A paired sample *t*-test was used to assess the differences between the investigated reference schemes. The statistical significance of *t*-values was assessed through the non-parametric permutation test implemented in the FieldTrip software package (Oostenveld et al., 2011). This approach aims at evaluating the *p*-values associated to the observed *t*-values by comparison with an empirical reference distribution constructed from data which do not violate the null hypothesis (Maris and Oostenveld, 2007). To this purpose, for each observed *t*-value from pairwise comparisons between the different reference schemes, say reference 1 vs. reference 2, we generated 10,000 random partitions of the data by randomly shuffling the two references in each subject. The paired sample *t*-test was then applied to each of the 10,000 random partitions. As the random partitions do not violate the null hypothesis by construction, the respective *t*-values provide the distribution for the test statistic under the null hypothesis. The *p*-value associated to the observed *t*-value was finally evaluated as the proportion of random partitions that resulted in *t*-values larger than the observed one in absolute value.

## 3. Results

### 3.1. Simulation results

The effects of the different reference schemes, i.e., Cz, DLM, AVE, REST_sph_, REST_std_, and REST_ind_, on the estimation of bicoherence, cross-bicoherence, and antisymmetric cross-bicoherence were assessed in terms of relative error (RE) between the estimates obtained from the datasets referenced to a point at infinity and those obtained from the re-referenced datasets, as defined in Equations (16–18). As the point at infinity behaves as an ideal neutral reference, the best reference scheme is the one that yields the smallest RE.

**Figure 1:**
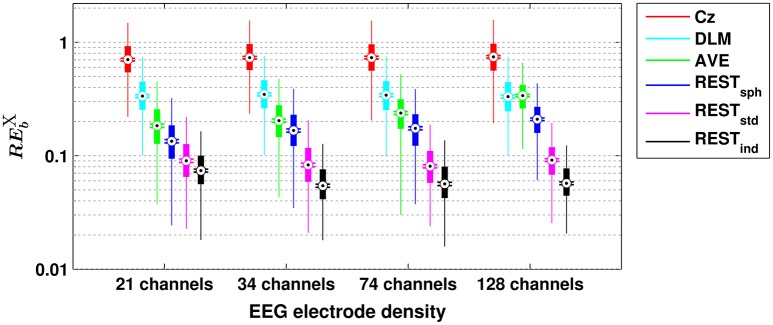
**Box plots for the relative error for bicoherence ($RE_{b}^{\text{X}}$) evaluated with different EEG reference schemes and with different EEG electrode densities**. The ordinate axis is logarithmically scaled. Each box plot displays the RE-values from 1,000 simulation repetitions.

The box plots in Figure 1 show the dependence of the RE for bicoherence on the reference scheme and on the EEG electrode density. In particular, each box plot displays the distribution of the RE-values obtained from the 1,000 simulation repetitions: the rectangular box denotes the range from the 25th to the 75th percentile; the whiskers extend 1.5 times this range, such that they roughly cover the 99.3% of the data in case of normal distribution; the black dot denotes the median value; the two horizontal lines denote the notches for assessing the significance of difference of medians, i.e., two medians are significantly different at the 5% level if their notch intervals do not overlap. It can be noted that, among the investigated reference schemes, the reference to Cz is the one that shows the largest RE, as demonstrated by the median values being larger than 70%. Lower values of RE can be achieved by using the DLM reference (i.e., median RE of about 34%), although these values are still larger than those obtained for the REST, or for the AVE reference (except for the case of 128 channels, when the median RE obtained from DLM and AVE do not show significant differences). Notably, the RE-values for Cz and DLM references are not noticeably affected by the EEG electrode density. The AVE reference performs better than the Cz and DLM references, as demonstrated by the RE being effectively reduced. Interestingly, the RE for AVE reference slightly increases with increasing electrode number (i.e., median RE of about 18% for 21 channels, 20% for 34 channels, 23% for 74 channels, and 34% for 128 channels). The REST performs better than all the other reference schemes, with the lowest RE being achieved when using a realistic individual head model and high density EEG (i.e., median RE of about 7% for 21 channels, and 5% for 34-, 74- and 128-channels). In general, the more realistic the head model is, the better the REST performance is. Indeed, even if a realistic standard model is used in place of a realistic individual model, the median RE is lower than 10%, regardless of the number of channels, while significantly larger errors occur if a spherical head model is used, especially for increasing electrode density. It must be noted, however, that even in the case of a spherical head model, the REST performance is better than the ones of AVE, DLM, or Cz.

**Figure 2:**
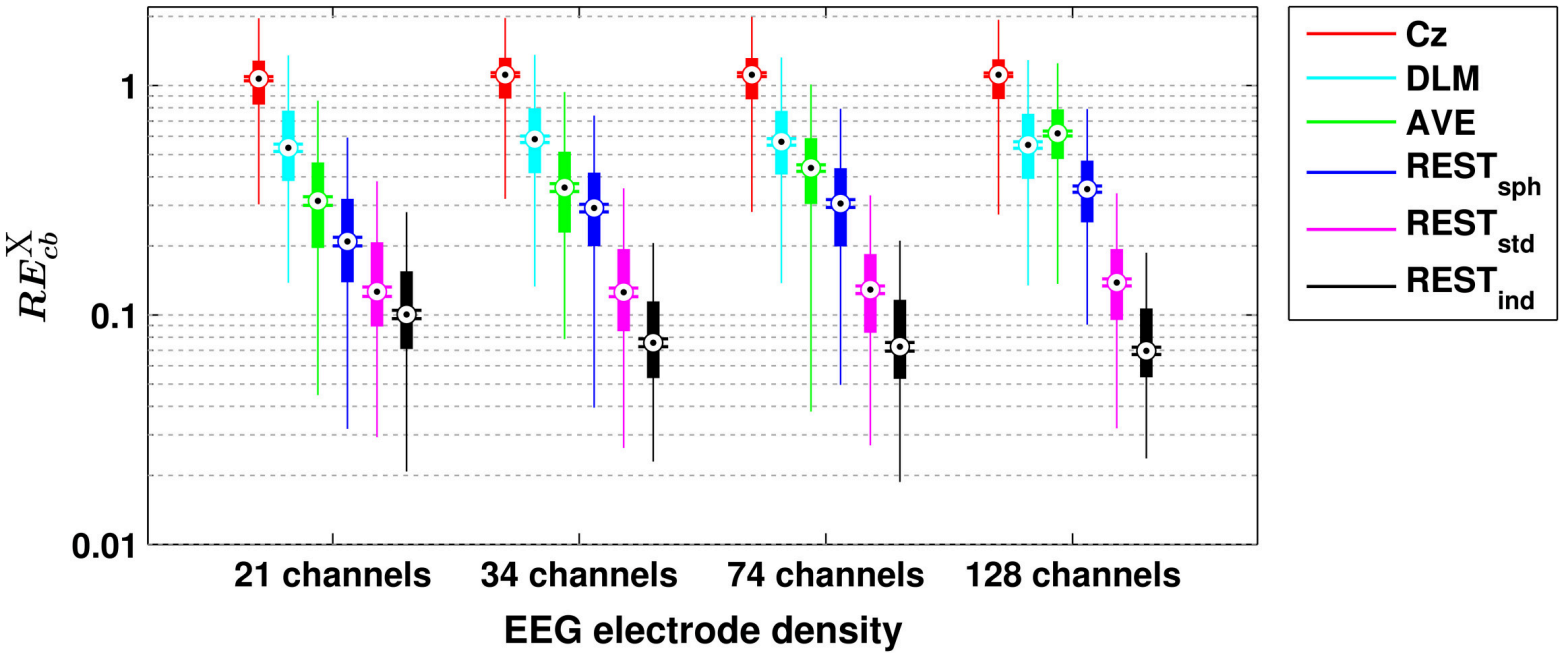
**Box plots for the relative error for cross-bicoherence ($RE_{cb}^{\text{X}}$) evaluated with different EEG reference schemes and with different EEG electrode densities**. The ordinate axis is logarithmically scaled. Each box plot displays the RE-values from 1,000 simulation repetitions.

**Figure 3:**
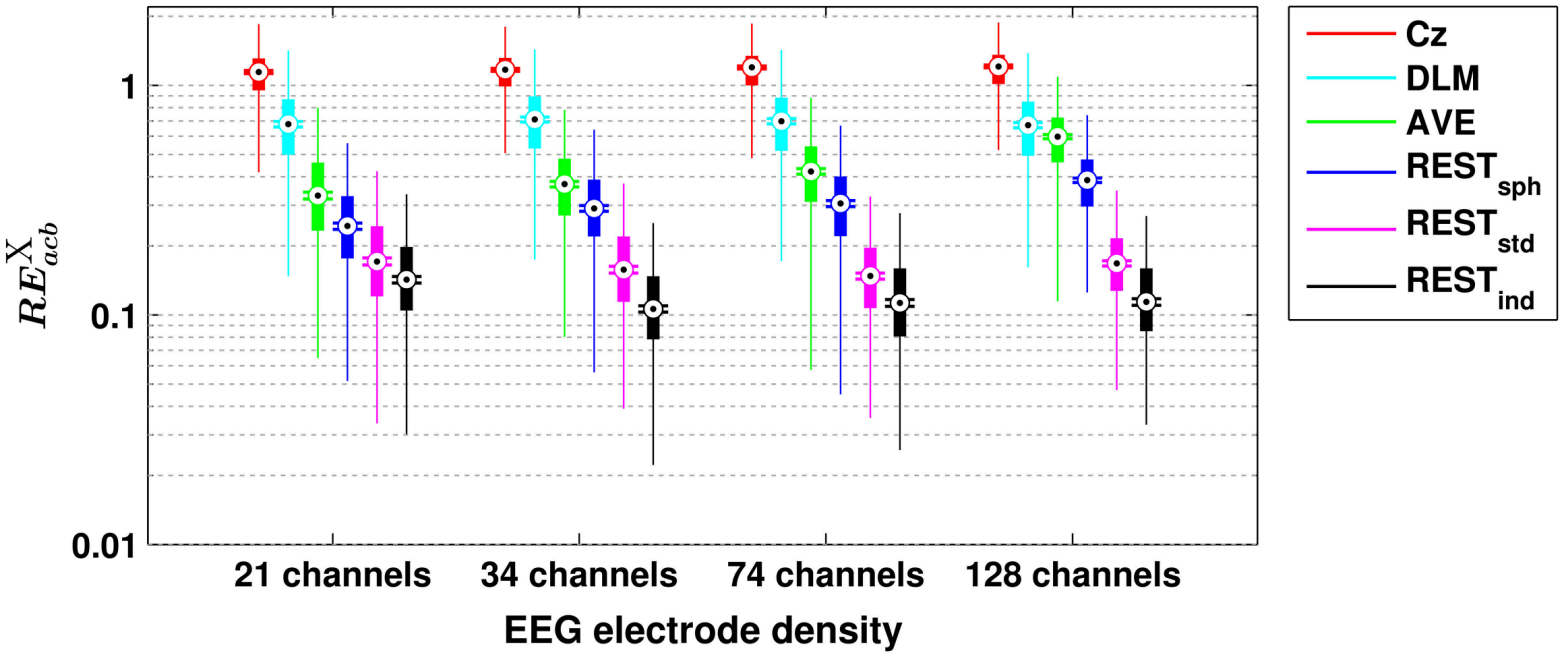
**Box plots for the relative error for antisymmetric cross-bicoherence ($RE_{acb}^{\text{X}}$) evaluated with different EEG reference schemes and with different EEG electrode densities**. The ordinate axis is logarithmically scaled. Each box plot displays the RE-values from 1,000 simulation repetitions.

A similar scenario can be found when assessing the performances of the different reference schemes in the estimation of scalp EEG connectivity based on either cross-bicoherence (Figure 2) or antisymmetric cross-bicoherence (Figure 3). Indeed, although a significant and systematic increase of median RE can be observed for both of the connectivity measures as compared to bicoherence (Figure 1), such an increase does not affect the contrast between the different EEG reference performances. In particular, the REST still remains the best choice of EEG reference scheme, especially when using a high density EEG system and a realistic individual head model (i.e., for cross-bicoherence: median RE of about 10% for 21 channels, and 7% for 34-, 74- and 128-channels; for antisymmetric cross-bicoherence: median RE of about 14% for 21 channels, and 11% for 34-, 74- and 128-channels). If the latter is not available, REST still performs better than the other reference schemes, but the dependence of RE on the EEG density is negligible (i.e., for REST_std_) or even the opposite (i.e., for REST_sph_). The AVE reference performs worse than REST (i.e., median RE > 30%), while the largest RE is obtained when using DLM (i.e., median RE > 50%) or Cz (i.e., median RE > 100%). Finally, in the comparison between the two connectivity measures, it can be noted that the median RE for antisymmetric cross-bicoherence is generally larger than the respective values for cross-bicoherence.

### 3.2. Real data results

#### 3.2.1. Bicoherence

The bicoherence was estimated for each channel and for each combination of frequencies (*f*_1_, *f*_2_) up to *f*_1_ + *f*_2_ = 40Hz. For all subjects, a prominent peak of bicoherence was found at around (*f*_1_, *f*_2_) = (10, 10Hz) (shown in Figure S1 of the Supplementary Material), which essentially means that the EEG signals in the alpha band (i.e., at *f*_1_ = *f*_2_ = 10Hz) have a strong non-linear coupling with their first harmonically related components in the beta band (i.e., at *f*_3_ = *f*_1_ + *f*_2_ = 20Hz). The analysis was then restricted to bicoherence at the individual frequency pair where such a peak occurred.

**Figure 4:**
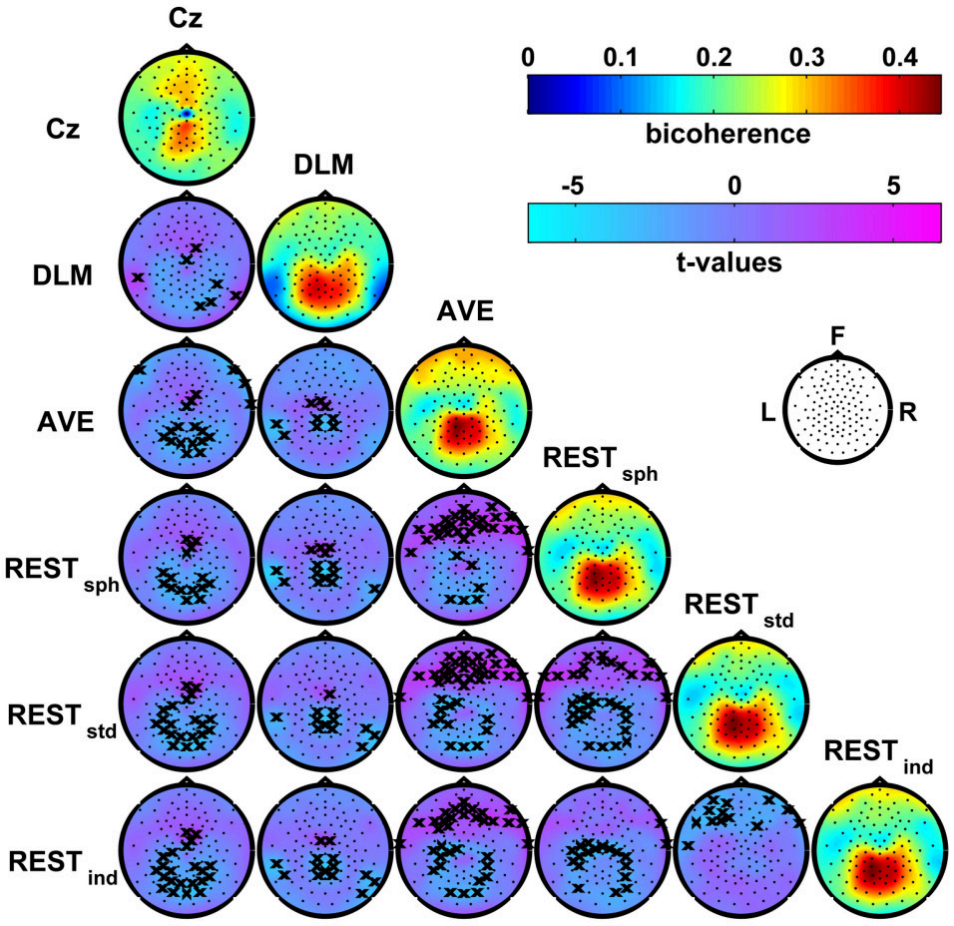
**Effects of the EEG reference choice on alpha-beta bicoherence patterns estimated from resting state EEG data**. Main diagonal: maps of the average bicoherence across subjects obtained for the different EEG reference schemes. Off-diagonal: maps of *t*-values for pairwise contrasts between bicoherence maps from different EEG reference schemes using a paired-sample *t*-test; the black crosses mark the channels showing significant differences at the *p* < 0.05 level (two-tailed) based on a permutation test (10,000 randomizations).

Figure 4 shows the patterns of bicoherence obtained for the different EEG reference schemes. In particular, the maps on the main diagonal show the average bicoherence across subjects obtained for each of the EEG reference. The off-diagonal maps show the *t*-values resulting from pairwise contrasts between bicoherence maps using a paired-sample *t*-test; here, the channels showing significant differences at the *p* < 0.05 level were marked with a cross. First, all the bicoherence maps reveal a strong level of bicoherence in a wide area roughly extending from the centro-parietal to the occipito-parietal channels. However, both the values and spatial distribution of bicoherence in this area change according to the chosen reference scheme. Second, there are a number of other significant differences in these maps, which can be better appreciated by looking at the *t*-value maps in the same figure. Indeed, the reference to Cz induces a systematic increase of bicoherence in the fronto-central channels as compared to all the other references. At the same time, Cz suppresses the values of bicoherence in proximity of the reference electrode and, if compared to AVE and REST reference, over the parietal regions. The DLM reference scheme is characterized by an overall increase of bicoherence in the central channels, and by a decrease of bicoherence in the parietal channels and in proximity of the left and right mastoids used for the reference signal. Although the bicoherence maps obtained with the AVE and the REST reference look similar based on a qualitative evaluation, our analysis highlighted systematic and significant differences in these patterns. In particular, the AVE reference causes an increase of bicoherence in the frontal channels as compared to REST, along with a decrease of bicoherence in the occipital and in some of the parietal and central channels. Finally, the bicoherence maps obtained with REST significantly change according to the head model used for the data standardization as revealed by the *t*-values, although the differences can be poorly appreciated from a visual contrast between these map.

**Figure 5:**
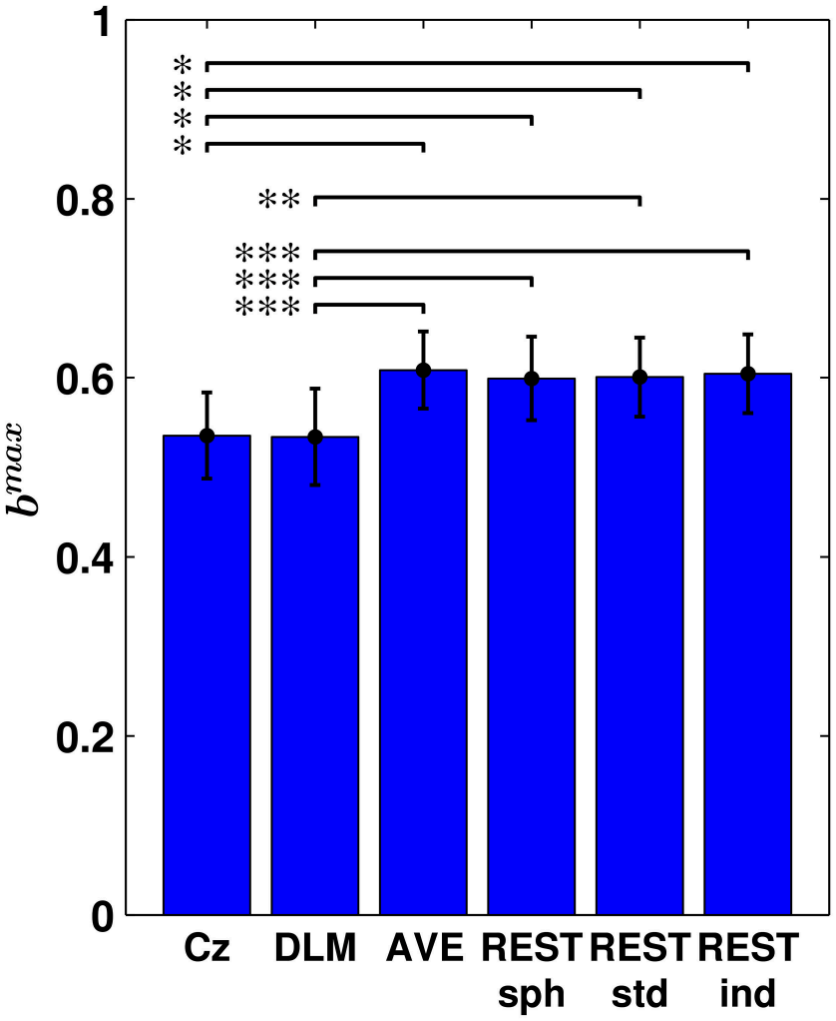
**Maximum bicoherence (***b***^***max***^) obtained for the different EEG reference schemes**. The bar plot shows the average *b*^*max*^ across subjects and its standard error. Statistical comparison between the different reference schemes was performed by using a paired sampled *t*-test (^*^p < 0.05; ^**^p < 0.01; ^***^p < 0.001; two-tailed; fdr-corrected; permutation test).

In order to asses whether the choice of the EEG reference affects a global measure of bicoherence, the maximum value of bicoherence over channels, i.e., *b*^*max*^, was considered for each subjects. The results are summarized in Figure 5, where the bar plot shows the average *b*^*max*^ across subjects and its standard error, in dependence of the reference scheme. Notably, this analysis revealed that the Cz and DLM references yield a significantly lower *b*^*max*^ than the AVE and REST references, with the statistical significance being determined by permutation testing.

#### 3.2.2. Non-linear connectivity analysis

Connectivity with respect to two seed channels located over the left and right medial-parietal areas, i.e., P1 and P2, was estimated using both the cross-bicoherence and the antisymmetric cross-bicoherence. The obtained results are summarized below.

**Figure 6:**
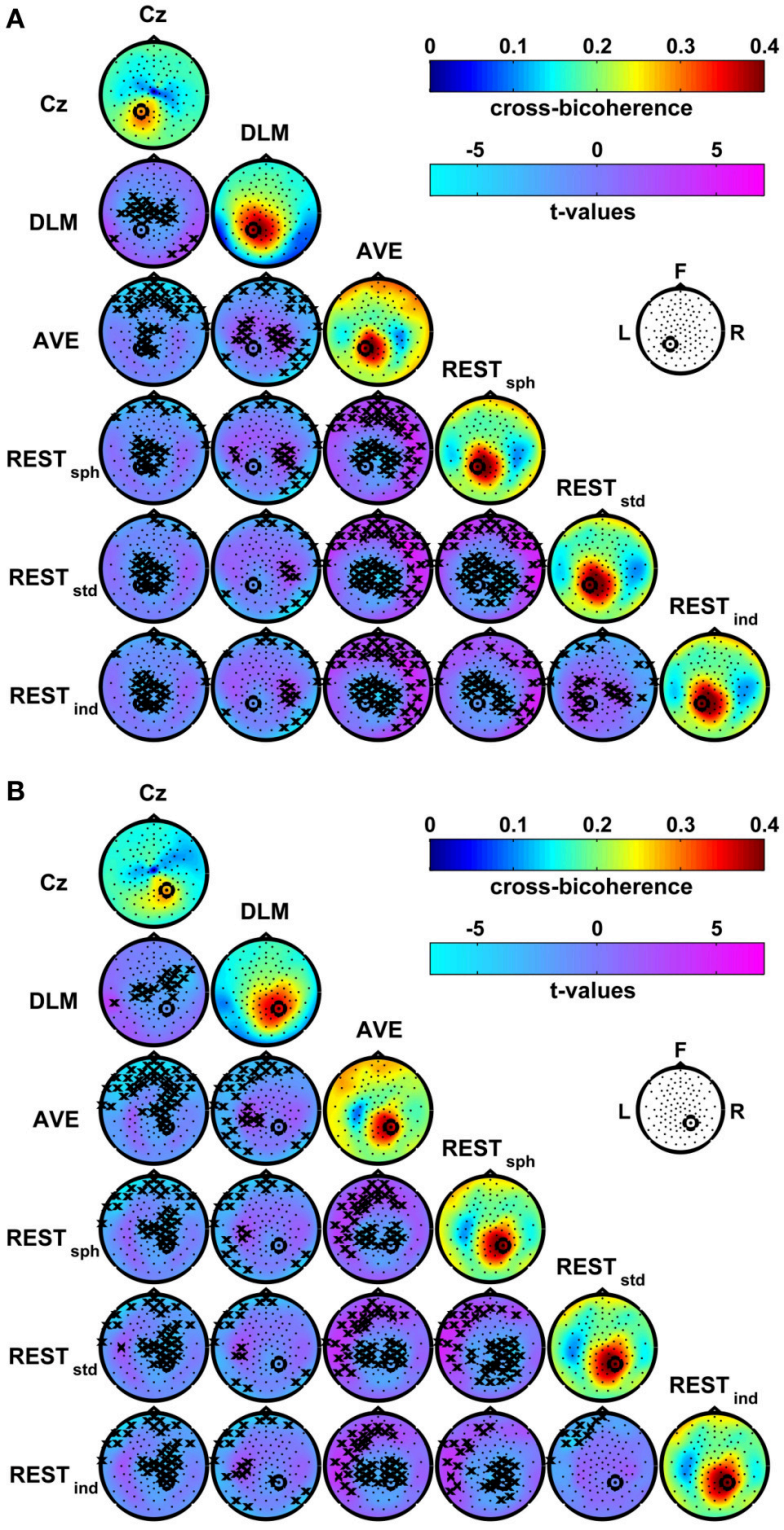
**Effects of the EEG reference choice on the estimation of alpha-beta connectivity using cross-bicoherence**. Panel **(A)**: maps of connectivity with seed channel P1. Panel **(B)**: maps of connectivity with seed channel P2. The seed channels have been marked by a black circle. Main diagonals: maps of the average cross-bicoherence across subjects obtained for the different EEG reference schemes. Off-diagonals: maps of *t*-values for pairwise contrasts between cross-bicoherence maps from different EEG reference schemes using a paired-sample *t*-test; the black crosses mark the channels showing significant differences at the *p* < 0.05 level (two-tailed) based on a permutation test (10,000 randomizations).

Figure 6 shows the group average maps of the cross-bicoherence with seed channels P1 (Figure 6A) and P2 (Figure 6B) for each of the EEG reference schemes. The *t*-value maps are also shown for the contrast between the different reference conditions. The cross-bicoherence maps reveal a main pattern of interaction where the seed channel is primarily coupled to its neighbor channels. Beside this finding, the pairwise contrasts between these maps reveal a number of significant differences which, as similarly discussed above for bicoherence mapping, are only due to the choice of the particular EEG reference scheme. In particular, the Cz reference shows lower connectivity with the central channels as compared to DLM, and with the central and frontal channels as compared to AVE and REST. The DLM reference shows lower connectivity with the frontal channels, but higher connectivity with the central channels as compared to AVE or REST. At the same time, the DLM reference suppresses the connectivity with the channels located over the left and right mastoids as compared to all the other references. In the comparison between AVE and REST, the AVE reference shows lower connectivity with central channels, and higher connectivity with the fronto-central channels or with the temporal channels controlateral to the seed. Finally, for the REST, the head model accuracy has a significant impact on the estimation of the connectivity patterns. Specifically, if a spherical model is used (i.e., REST_sph_) instead of a realistic one (i.e., REST_std_ or REST_ind_), the connectivity with the central channels and with the channels located in proximity of the seed is lower, whilst the connectivity with the fronto-temporal and temporal channels controlateral to the seed is higher. A few significant differences also arise in REST_std_ as compared to REST_ind_, consisting in a lower connectivity with the frontal channels and, only for the connectivity with P1, in higher connectivity with the centro-parietal channels.

**Figure 7:**
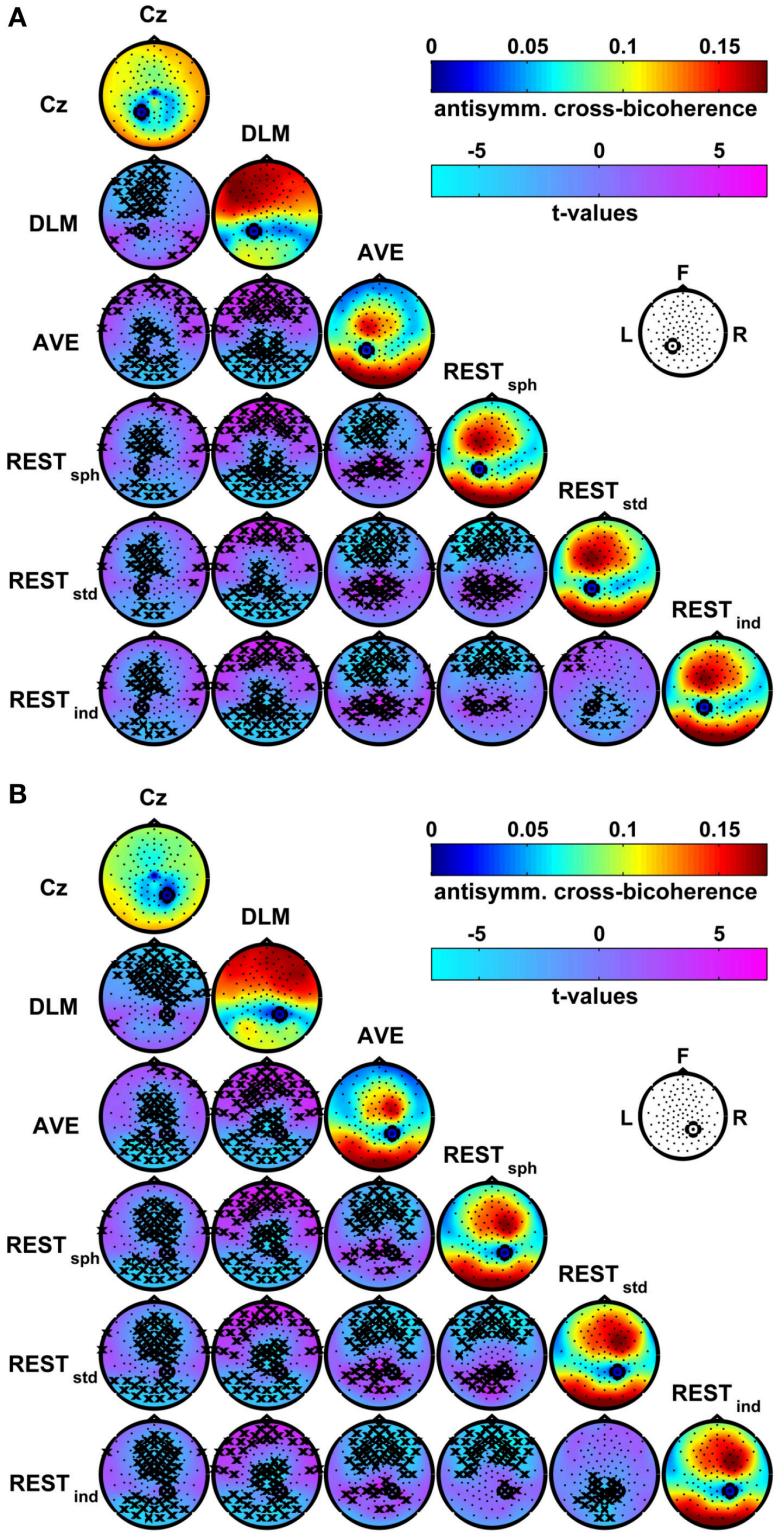
**Effects of the EEG reference choice on the estimation of alpha-beta connectivity using the antisymmetric cross-bicoherence**. Panel **(A)**: maps of connectivity with seed channel P1. Panel **(B)**: maps of connectivity with seed channel P2. The seed channels have been marked by a black circle. Main diagonals: maps of the average antisymmetric cross-bicoherence across subjects obtained for the different EEG reference schemes. Off-diagonals: maps of *t*-values for pairwise contrasts between antisymmetric cross-bicoherence maps from different EEG reference schemes using a paired-sample *t*-test; the black crosses mark the channels showing significant differences at the *p* < 0.05 level (two-tailed) based on a permutation test (10,000 randomizations).

A different pattern of interaction arises when the connectivity is estimated by using the antisymmetric cross-bicoherence. Figure 7 shows the maps of the antisymmetric cross-bicoherence with P1 (panel A) and P2 (panel B) obtained for the different EEG reference schemes and their respective contrasts. Notably, contrarily to the cross-bicoherence, the antisymmetric cross-bicoherence reveals a pattern of long range interaction between channels, which clearly results from this measure being not biased by the artifacts of EEG volume conduction. Moreover, large differences can be observed in the comparison between the different reference schemes, especially when contrasting Cz and DLM to AVE and REST. Indeed, for the Cz reference, the interaction is mainly with the occipital channels. For the DLM reference, the interaction is mainly with the frontal and central channels in the hemisphere ipsilateral to the seed. On the other hand, the AVE and REST references reveal a clear pattern of interaction of the seed channels, which we recall to be located in the medial-parietal left and right areas, with the channels in the fronto-central area ipsilateral to the seed and with channels in the left and right occipital areas. As discussed above for bicoherence and cross-bicoherence mapping, the *t*-value maps in Figure 7 reveal significant differences in the antisymmetric cross-bicoherence depending on the reference scheme, with specific spatial topographies. In particular, the Cz reference mainly shows a lower interaction with the frontal and fronto-central channels as compared to DLM, and with the fronto-central and occipital channels as compared to AVE and REST. The DLM shows larger connectivity with the frontal channels, and lower connectivity with the parietal and occipital channels as compared to AVE and REST. As compared to REST, the AVE reference shows lower connectivity with the frontal channels, and higher connectivity with the parietal and occipital channels. A similar distortion can be observed when contrasting REST_sph_ and REST_std_ or REST_ind_. Finally, the differences between REST_std_ and REST_ind_ are mainly located in the parietal and occipital channels, with the REST_std_ showing lower connectivity than REST_ind_.

## 4. Discussion

In the present study, the effects of four commonly used EEG reference schemes, i.e., Cz, DLM, AVE, and REST, on bispectral measures derived from EEG signals were investigated. To this purpose, the following bispectral measures were considered: (i) the bicoherence, which is a measure of the local degree of non-linear coupling within each of the EEG channels, reflecting the non-linear and non-Gaussian features of the underlying brain processes (Sigl and Chamoun, 1994; Bullock et al., 1997; Schack et al., 2002); and (ii) the cross-bicoherence and the antisymmetric cross-bicoherence, which are both measures of non-linear cross-frequency connectivity between different EEG channels, possibly reflecting long-range non-linear synchronization between neuronal populations (ShilS et al., 1996; Schack et al., 2002; Isler et al., 2008; Jirsa and Müller, 2013; Chella et al., 2016b). Particularly relevant for the estimation of connectivity from scalp EEG data is the antisymmetric cross-bicoherence, which, as opposed to the cross-bicoherence, is not biased by the artifacts due to volume conduction (Chella et al., 2014, 2016b).

The reference effects were first assessed by using simulations, where the above mentioned reference schemes were compared to the ideal case of the reference to a true neutral location, i.e., a point located at infinity (Kayser and Tenke, 2010; Nunez, 2010). In particular, the simulations examined the accuracy in estimating the bispectral measures in relation to EEG electrode density and, since the REST requires the solution of the EEG forward problem, to the head model accuracy. Notably, previous studies investigated the effects of the electrode density on the analysis of EEG potentials or power by using different reference schemes (Nunez and Srinivasan, 2006). Yao (2001), Zhai and Yao (2004), and Liu et al. (2015) also highlighted the importance of using an accurate head model as a key factor to improve the performance of REST. Marzetti et al. (2007), Qin et al. (2010), and Chella et al. (2016a) performed a comparative assessment of different EEG reference schemes in the estimation of linear scalp EEG connectivity based on coherency or imaginary part of coherency, demonstrating the validity of REST in data re-referencing. Along this line, this simulation study expands upon these previous findings by characterizing the effects of the reference choice on bispectrum-based non-linear EEG measures, an issue which is particularly relevant for several of applications, especially clinical, relying on bispectral analysis of the EEG (Freye and Levy, 2005; Mormann et al., 2005; Chua et al., 2009; Pritchett et al., 2010; Hayashi et al., 2014).

Our simulations showed that, as compared to all the other reference schemes, the reference to the physical electrode Cz induces the largest distortion in the estimates of bicoherence, cross-bicoherence, and antisymmetric cross-bicoherence. This is conceivably due to the reference electrode being in a location, i.e., the vertex, which is highly contaminated by the electrical activity of brain sources. Even though the mastoids are often regarded to as electrically inactive locations, the distortion induced by the DLM reference is also substantially large. Notably, the performances of the Cz and DLM references are not affected by the EEG electrode density, proving that indeed the observed distortion is only due to the electrical contamination of the reference signal. These results are consistent with previous findings (Dien, 1998; Hagemann et al., 2001; Nunez and Srinivasan, 2006; Chella et al., 2016a) and are particularly relevant for those studies using linked mastoids or a single cephalic electrode as a reference scheme for bicoherence and cross-bicoherence estimation (e.g., Hagihira et al., 2002; Schack et al., 2002; Hagihira et al., 2002; Schack et al., 2005).

Overall, the AVE reference provides better results as compared to Cz and DLM, although it is not completely free of biases. Most notably, the distortion induced by the AVE reference increases for increasing electrode density. This effect was already observed by Chella et al. (2016a) in the estimates of EEG imaginary coherency, thus confirming that an increased electrode density may be not a key factor to improve the performance of the AVE reference. Indeed, it is well-known that the actual accuracy of the AVE reference in approximating a theoretical zero-potential reference depends not only on the electrode density, but also on the electrode scalp coverage, i.e., which is limited to the upper part of the head (Tomberg et al., 1990; Dien, 1998; Nunez, 2010) or, as recently shown by Yao (2017), even on the head geometry.

The REST reference significantly reduces the above reference-induced distortion, with median values for relative errors being around 5% for the bicoherence, 7% for the cross-bicoherence, and 11% for the antisymmetric cross-bicoherence if a realistic individual head model and more than 34 EEG channels are used. In line with previous findings (Zhai and Yao, 2004; Liu et al., 2015; Chella et al., 2016a), this study shows that an accurate knowledge of the head model is crucial to improve the performance of REST standardization. Indeed, the distortion substantially increases if the head model is not sufficiently accurate, i.e., when using a realistic standard or spherical head model. However, it must be noted that, for a given sensor density, the REST still remains better choice than all the other reference schemes. In addition, these results demonstrate that the REST benefits from high density EEG only if used in combination with a realistic individual head model, whilst, if used in combination with a realistic standard or spherical head model, the effects of an increased electrode density are negligible or even the opposite.

The analysis of real EEG data provided further evidence of the reference effects on bispectral measures derived from EEG signals. This analysis was primarily focused on the contrast between the patterns of alpha-beta bicoherence as well as of bispectrum-based alpha-beta connectivities obtained from resting state EEG data with the different reference schemes. The results show that, indeed, there are systematic and significant differences in these patterns, which only depend on the use of the chosen reference scheme. In particular, the differences are larger for the Cz reference as compared to all the other reference schemes. This is conceivably due to the fact that the alpha and beta rhythms in the resting state EEG have a dominant activity in the occipito-parietal and central areas, which are in close proximity to the reference electrode. Substantial differences were also observed for DLM as compared to AVE and REST, while it should be noted that the differences between the AVE and REST are considerably small, although statistically significant. As for the analysis of connectivity patterns, this study shows that the antisymmetric bicoherence in combination with REST can provide patterns of long-range connectivity which can be directly interpreted in terms of functional interactions between the underlying brain sources. In particular, our findings (Figure 7) show an alpha-beta interaction between the left and right medial-parietal cortices with the ipsilateral frontal cortices, as well as with bilateral occipital cortices. Indeed, there is abundant evidence of this frequency specific signature of occipito-parietal and frontal areas in the resting state (Palva et al., 2005; Nikulin and Brismar, 2006; Sauseng and Klimesch, 2008; Marzetti et al., 2013; Hillebrand et al., 2016; Siebenhühner et al., 2016). It is thus conceivable that a circuit comprising occipito-parietal and frontal areas is recruited through an alpha-beta cross-frequency synchronization mechanism. The above findings could not be argued from the analysis of cross-bicoherence (Figure 6) which, in fact, seems to be mainly biased by volume conduction effects.

Besides the methods concerned in this paper, when dealing with the issue of the EEG reference, the possibility of getting rid of the reference effects by performing the analysis at the source level should also be considered. Indeed, it has been shown that the choice of the EEG reference does not affect the reconstruction of neural active sources, at least for noiseless potentials (Pascual-Marqui and Lehamann, 1993; Geselowitz, 1998). Thus, once a solution to the EEG inverse problem has been provided, bispectral analysis can be performed directly on reconstructed source time courses. However, in practice this approach still depends on a number of factors including, e.g., the accuracy in the knowledge of the head model, the EEG sensor density, or the choice of the inverse solver, which may affect the accuracy of source reconstruction. The advantages and disadvantages of source-level analysis over sensor-level analysis will not be addressed here, as they go beyond the scope of this work. The aim of our study was to show how the choice of the EEG reference affects bispectral analysis of sensor-level EEG data, which is a standard practice for many research or clinical applications (e.g., Chua et al., 2009; Pritchett et al., 2010; Hayashi et al., 2014; Chella et al., 2016b; Özkurt, 2016).

In conclusion, the present study provides evidence that, also in the analysis of non-linear features of EEG signals and interactions, the choice of the reference may significantly affect the study results and the derived conclusions. To minimize this effect, we recommend the use of the REST reference, which guarantees less biased results and a straightforward comparison across different laboratories or databases, with a clear impact for research and clinical practice.

## Author contributions

FC, VP, and LM conceived and designed the study. FC acquired the data and performed the analysis. All the authors interpreted the results. FC, AD, and LM wrote the manuscript. All the authors critically reviewed the manuscript.

## Funding

The author FC has received funding from the European Commission Horizon 2020 research and innovation program under Grant Agreement No. 686865 (BREAKBEN—H2020-FETOPEN-2014-2015/H2020-FETOPEN-2014-2015-RIA). The content reflects only the author's view and the European Commission is not responsible for the content. This work was partially supported by the University of Chieti-Pescara Faculty Resources Grant 2016 of author LM, entitled “Methods for the study of functional connectivity with MEG and EEG and applications to neuroscience.”

### Conflict of interest statement

The authors declare that the research was conducted in the absence of any commercial or financial relationships that could be construed as a potential conflict of interest.
