# Supplementary material for: Non-linear Analysis of Scalp EEG by Using Bispectra: The Effect of the Reference Choice
Source: Front Neurosci. 2017 May 16;11:262. doi: 10.3389/fnins.2017.00262 (PMC5432555; doi:10.3389/fnins.2017.00262)
Supplement: Supplementary file 1 [file Image1.pdf]

Supplementary Material: Non-linear Analysis of Scalp EEG by Using Bispectra: The Effect of the Reference Choice

Federico Chella [1,2,*], Antea D'Andrea [1,2], Alessio Basti [1,2], Vittorio Pizzella [1,2] and Laura Marzetti [1,2,*]
*Correspondence:
Federico Chella
federico.chella@unich.it

Laura Marzetti
laura.marzetti@unich.it

1 SUPPLEMENTARY FIGURE

Figure S1 shows the plots of bicoherence as a function of frequencies f_1 and f_2 and for each of the 10 subjects participating to the real data experiment. In particular, this figure represents the results obtained from the datasets re-referenced to $\mathrm{REST}_{\mathrm{ind}}$. Similar results (not shown here) were obtained for all the other reference schemes. Note that the bicoherence was estimated for each of the N channels and, thus, N estimates were obtained for each frequency pair. However, only the maximum over these N estimates can be appreciated from these plots. A common feature of all subjects is a prominent peak of bicoherence at around $(f_1, f_2) = (10\ \mathrm{Hz}, 10\ \mathrm{Hz})$, which reflects a nonlinear coupling between EEG signal components in the alpha ($f_1 = f_2 = 10$ Hz) and beta ($f_3 = f_1 + f_2 = 20$ Hz) bands.

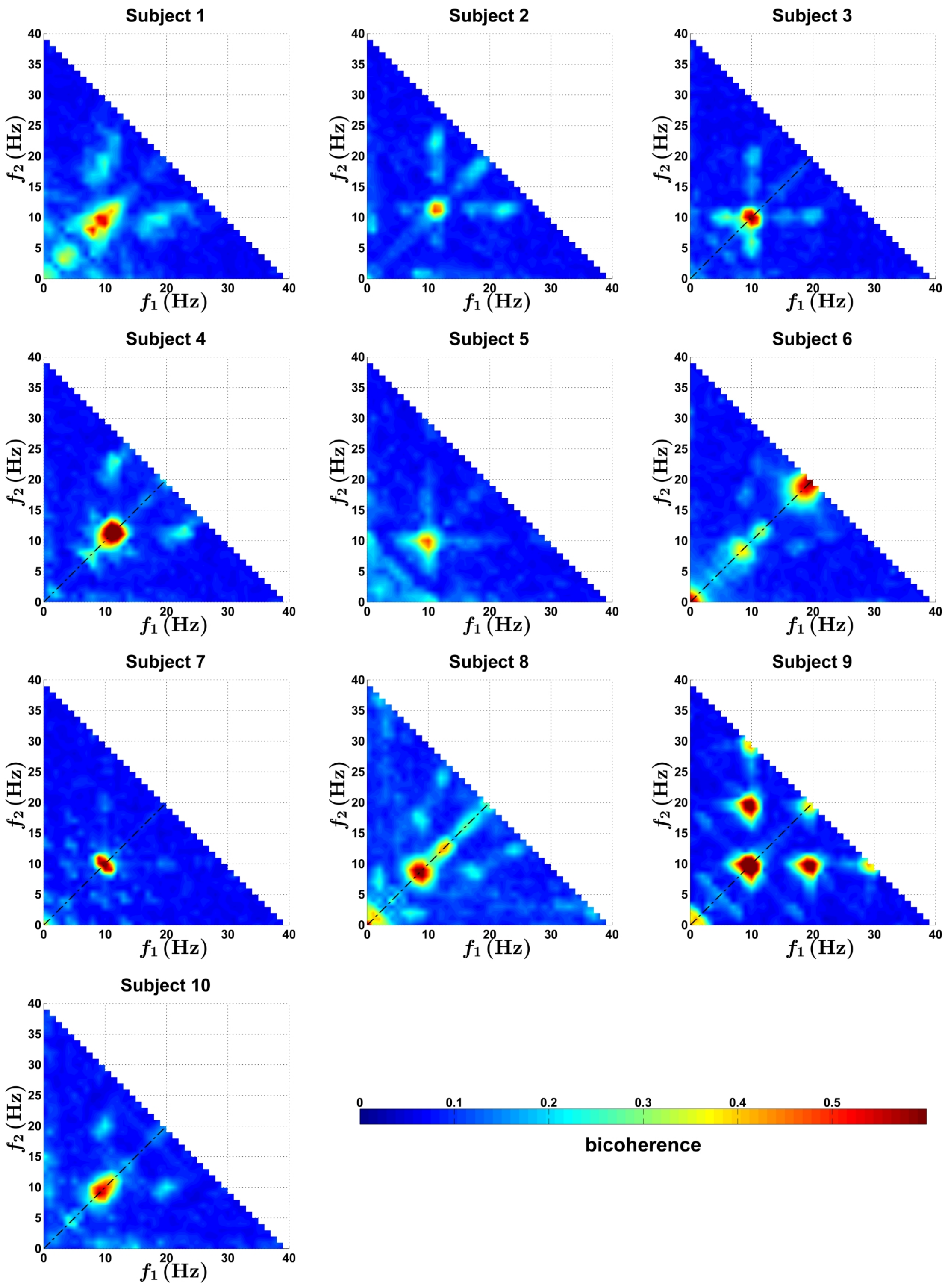

Figure S1. Plots of bicoherence as a function of frequencies f_1 and f_2 and for each of the 10 subjects participating to the real data experiment. These plots were obtained from the dataset re-referenced to $\mathrm{REST_{ind}}$.
